# Comparison of Mineral Element Content in a Functional Food Maca (*Lepidium meyenii* Walp.) from Asia and South America

**DOI:** 10.1155/2015/530541

**Published:** 2015-07-08

**Authors:** Ji Zhang, Han-Mo Wang, Yan-Li Zhao, Zhi-Tian Zuo, Yuan-Zhong Wang, Hang Jin

**Affiliations:** Institute of Medicinal Plants, Yunnan Academy of Agricultural Sciences, Kunming 650200, China

## Abstract

Contents of eight mineral elements in maca (*Lepidium meyenii* Walp.) from China and Peru were determined by inductively coupled plasma optical emission spectroscopy. Cu contents in maca samples from China (2.5–31 mg kg^−1^ dry weight, dw) were higher than the samples from Peru (<2.1 mg kg^−1^ dw). Na in two samples from China was found to be significantly of high content (2400 and 2600 mg kg^−1^ dw). The contents (mg kg^−1^ dw) of B, Co, Cr, Li, Ni, and Zn were, respectively, 8.1–21, <0.023, <1.1~3.5, 0.020–0.17, 0.085–4.5, and 10–39 for the samples from China, while being 6.6–12, <0.023, <1.1~2.3, 0.035–0.063, 0.68–1.7, and 27–39 for the samples from Peru.

## 1. Introduction

Maca (*Lepidium meyenii* Walp.) is an endemic highland crop of the Central Andes which is grown from central Peru to Bolivia and northwestern Argentina [1, 2]. This plant has great potential as an adaptogen and appears to be promising as a nutraceutical in the prevention of several diseases [3]. Maca roots have been traditionally used to increase the rate of fertility in both humans and livestock [4]. Over the past 20 years, interest in maca has increased in many parts of the world [5]. Commercial maca products have gained popularity as dietary supplements for aphrodisiac purposes and for increasing fertility and stamina [6]. Recently, maca industry develops fast in Yunnan Province, China. In Yunnan, until the end of 2010, maca growing and promotion areas reached 175 hm^2^ and maca yield achieved 780 t, taking up to 90% and 93%, nationwide [7].

Mineral elements in food are very important because the quality of many functional foods and medicines depends on the content and type of minerals [8]. In the US, national surveys show that micronutrient inadequacies are widespread and mineral supplement helps fulfill micronutrient requirements in adults and children [9]. Up to now, only limited studies on selected elements in maca from different origins have been carried out [10, 11]. Moreover, in some studies, no reference material had been certified for elemental analysis, so there has been doubt about accuracy of the determination.

In the present study, inductively coupled plasma optical emission spectroscopy (ICP-OES) was used to determine the contents of eight elements (B, Co, Cr, Cu, Li, Na, Ni, and Zn) in maca samples, and a comparison was made between the samples from China and Peru.

## 2. Materials and Methods

All reagents used in the present work were of analytical reagent grade. Maca sample digests were prepared using HNO_3_ and H_2_O_2_ of ultrapure grade. Standard sample solutions of B, Co, Cr, Cu, Li, Na, Ni, and Zn obtained from Standard Material Center of China were used to make a mixed calibration curve in the range of 0–400 *μ*g mL^−1^.

Ten maca samples were collected from four places of Yunnan, China, and four samples were from Peru. These samples were washed with deionized water thoroughly, dried to a constant weight, grounded into powder using an agate mortar, passed through a 60-mesh sieve, and stored in the plastic bags. 500 mg of each maca sample was weighed into an acid washed Teflon digestion tube. 8 mL of HNO_3_, 2 mL of H_2_O_2_, and 1 mL ultrapure water were added to the vessel.

Samples were digested in a microwave dissolver (Ethos One, Milestone, Italy) equipped with PTFE vessels. A three-step program was applied to the samples (Table 1). The extract was transferred into a cuvette and made up to 25 mL with ultrapure water.

Simultaneous multielement detection of B, Co, Cr, Cu, Li, Na, Ni, and Zn was performed with ICP-OES (ICPE-9000, Shimazu, Japan). The optimal instrumental conditions for ICP-OES are shown in Table 2. The emission wavelengths and detection limits for each element are shown in Table 3. Every sample was measured with three replicates. Blank experiments were prepared in the same way.

To ensure the precision of the experiment, the certified reference material GBW10028 (dried herb powder* Astragalus membranaceus*) was used for validation of the method (Table 4). The relative standard deviation (RSD) was found to be below 8%, and the recoveries range from 92% to 109%, which proved that this method was accurate and precise.

## 3. Results and Discussion

Contents of B, Co, Cr, Cu, Li, Na, Ni, and Zn in maca samples are shown in Table 5. All element contents were determined on a dry weight basis.

Boron is essential for humans. Boron affects fat and lipid metabolism, minerals and mineral metabolism, vitamin D, and bone development [12]. Our B values in maca from China and Peru were 8.1–21 mg kg^−1^ dw and 6.6–12 mg kg^−1^ dw, respectively, which were in agreement with that reported in the literature (8.8 mg kg^−1^ dw) [13].

Cobalt is an essential micronutrient in the form of vitamin B_12_, but cobalt is toxic in larger doses or long-term exposure at a low level. The adverse effects of cobalt relate to various organs and tissues and may include a possible carcinogenic potential [14]. Chromium can induce the oxidative stress and genotoxicity in chromium exposed population [15]. Lithium appears to play an especially important role during the early fetal development [16]. The contents (mg kg^−1^ dw) of Co, Cr, and Li were, respectively, <0.023, <1.1–3.5, and 0.020–0.17 for the samples from China and <0.023, <1.1–2.3, and 0.035–0.063 from Peru. Up to now, there was no other report on the contents of Co, Cr, and Li in maca.

Copper plays role as a cofactor for numerous enzymes in humans. Its compounds show vast array of biological actions, such as anti-inflammatory, antiproliferative, and biocidal activities [17]. Cu contents in the samples from China (2.5–31 mg kg^−1^ dw) were higher than the samples from Peru (<2.1 mg kg^−1^ dw). Published data available on Cu in maca from China were 4.0–32 mg kg^−1^ dw [10, 11, 13, 18], while those from Peru were 1.5–62 mg kg^−1^ dw [10, 11, 19–22].

Sodium is an essential element. In mammals, sodium concentrations in blood are held high by strict homeostatic mechanisms [23]. Na in the samples from China was in the range <30–2600 mg kg^−1^ dw which is similar to the value (67–2400 mg kg^−1^ dw) in maca from China in literatures [11, 13, 18]. However, our data on Na in the samples from Peru (<30–41 mg kg^−1^ dw) are lower than the values reported (110–190 mg kg^−1^ dw) in the literatures [11, 19–22].

Nickel compounds have showed an increased risk of lung and nasal cancer in humans [24]. The contents of Ni in the samples were 0.085–4.5 mg kg^−1^ dw from China and 0.68–1.7 mg kg^−1^ dw from Peru. However, higher value (11.3 mg kg^−1^ dw) was found in the literature on the maca from China [13].

Zinc is essential for a number of physiological functions and plays a significant role in many enzyme actions in the living systems [25]. Zn contents in samples were found to be 19–39 mg kg^−1^ dw from China and 27–39 mg kg^−1^ dw from Peru, which were in accordance with the values for maca in literature available (25–89 mg kg^−1^ dw from China and 16–58 mg kg^−1^ dw from Peru) [10, 11, 13, 18–22].

## 4. Conclusions

The contents of eight elements of maca collected from China and Peru were determined. Cu contents in all of the maca samples from China, as well as Na contents in two samples from China, were remarkably higher than those values in other samples.

## Figures and Tables

**Table 1:** Microwave digestion parameters.

Step	Power (W)	Rising temperature time (min)	Temperature (°C)	Running time (min)
1	1800	5	120	5
2	1800	5	170	10
4	1800	5	180	55

**Table 2:** Operating conditions of ICP-OES.

Instrument	ICPE-9000
Spectrometer	Echelle grating
	CCD detector

Power	1.20 kW

Output power	1.2 kW

Argon flow	Cooling gas: 10 L/min
	Auxiliary: 0.6 L/min
	Carrier gas: 0.8 L/min

Nebuliser	Pneumatic (glass concentric)

Spray chamber	Glass cyclonic

Plasma viewing	Axial

Replicates for each analysis run	3

Sample uptake delay	30 s

**Table 3:** Wavelength and the detection limit for elemental analysis.

Element	Wavelength (nm)	Detection limits (mg kg^−1^)
B	249.773	1.135
Co	228.616	0.023
Cr	205.552	1.089
Cu	224.700	2.085
Li	670.784	0.0146
Na	330.232	29.95
Ni	231.604	0.0585
Zn	213.856	0.1595

**Table 4:** Determined and certified values of elements in GBW10028 (*Astragalus membranaceus*), *n* = 4.

Element	Certified value (mg kg^−1^)	Determined value (mg kg^−1^)	Recovery (%)
B	16.8 ± 1.6	18.3 ± 0.8	108.9
Co	0.44 ± 0.03	0.43 ± 0.02	97.7
Cr	2.2 ± 0.4	2.3 ± 0.5	103.2
Cu	8.5 ± 0.7	8.2 ± 0.7	97.0
Li	1.25 ± 0.12	1.15 ± 0.04	92.0
Na	1460 ± 190	1570 ± 46	107.5
Ni	2.26 ± 0.15	2.22 ± 0.01	98.3
Zn	22.3 ± 1.0	23.0 ± 1.5	103.1

**Table 5:** Element contents of maca samples, *n* = 3.

Number	Site	Element (mg kg^−1^ dw)							
		B	Co	Cr	Cu	Li	Na	Ni	Zn
1	Shangri-la, China	13 ± 2	nd	1.4 ± 0.8	2.5 ± 1.4	0.028 ± 0.014	nd	1.4 ± 0.1	20 ± 2
2	Shangri-la, China	12 ± 1	nd	3.0 ± 2.1	7.6 ± 3.3	0.11 ± 0.02	31 ± 23	2.0 ± 0.2	25 ± 3
3	Shangri-la, China	13 ± 1	nd	1.9 ± 1.3	5.7 ± 4.2	0.047 ± 0.028	170 ± 60	0.85 ± 0.61	23 ± 1
4	Zhaotong, China	9.0 ± 1.0	nd	2.5 ± 1.8	8.1 ± 3.6	0.051 ± 0.011	190 ± 180	1.5 ± 0.9	19 ± 2
5	Zhaotong, China	20 ± 1	nd	1.4 ± 0.8	6.1 ± 1.6	0.17 ± 0.02	2600 ± 50	0.93 ± 0.21	34 ± 1
6	Zhaotong, China	21 ± 1	nd	nd	9.3 ± 1.7	0.17 ± 0.01	2400 ± 300	1.7 ± 0.4	33 ± 2
7	Dongchuan, China	12 ± 0.4	nd	3.5 ± 2.0	7.4 ± 0.9	0.085 ± 0.009	59 ± 28	1.0 ± 0.1	29 ± 1
8	Dongchuan, China	9.7 ± 0.3	nd	nd	12 ± 1	0.043 ± 0.092	100 ± 60	1.5 ± 0.4	29 ± 0.4
9	Lijiang, China	8.1 ± 0.4	nd	1.1 ± 0.9	6.9 ± 2.2	0.020 ± 0.010	nd	1.2 ± 0.3	22 ± 1
10	Lijiang, China	13 ± 1	nd	1.6 ± 0.9	31 ± 3	0.16 ± 0.09	43 ± 25	4.5 ± 0.5	39 ± 4
11	Peru	11 ± 0.1	nd	nd	nd	0.050 ± 0.010	37 ± 2	1.4 ± 0.8	27 ± 1
12	Peru	6.6 ± 0.1	nd	nd	nd	0.063 ± 0.010	nd	1.7 ± 0.02	35 ± 2
13	Peru	11 ± 0.1	nd	2.1 ± 1.7	nd	0.040 ± 0.010	41 ± 37	0.68 ± 0.14	34 ± 1
14	Peru	12 ± 0.2	nd	2.3 ± 2.1	nd	0.035 ± 0.009	nd	0.87 ± 0.09	39 ± 2

Note: samples 1–10 were from China, while samples 11–14 were from Peru. “nd” means not detected.
